# Using Functional Near Infrared Spectroscopy (fNIRS) to Study Dynamic Stereoscopic Depth Perception

**DOI:** 10.1007/s10548-016-0476-4

**Published:** 2016-02-22

**Authors:** Laura M. Ward, Gordon Morison, William A. Simpson, Anita J. Simmers, Uma Shahani

**Affiliations:** Department of Vision Sciences, Glasgow Caledonian University, 70 Cowcaddens Road, Glasgow, G4 0BA UK; Department of Engineering, Glasgow Caledonian University, 70 Cowcaddens Road, Glasgow, G4 0BA UK; School of Psychology, Plymouth University, Drake Circus, Plymouth, Devon PL4 8AA UK

**Keywords:** fNIRS, Depth perception, Random dot stereogram, Binocular disparity, Haemodynamic response

## Abstract

The parietal cortex has been widely implicated in the processing of depth perception by many neuroimaging studies, yet functional near infrared spectroscopy (fNIRS) has been an under-utilised tool to examine the relationship of oxy- ([HbO]) and de-oxyhaemoglobin ([HbR]) in perception. Here we examine the haemodynamic response (HDR) to the processing of induced depth stimulation using dynamic random-dot-stereograms (RDS). We used fNIRS to measure the HDR associated with depth perception in healthy young adults (n = 13, mean age 24). Using a blocked design, absolute values of [HbO] and [HbR] were recorded across parieto-occipital and occipital cortices, in response to dynamic RDS. Control and test images were identical except for the horizontal shift in pixels in the RDS that resulted in binocular disparity and induced the percept of a 3D sine wave that ‘popped out’ of the test stimulus. The control stimulus had zero disparity and induced a ‘flat’ percept. All participants had stereoacuity within normal clinical limits and successfully perceived the depth in the dynamic RDS. Results showed a significant effect of this complex visual stimulation in the right parieto-occipital cortex (p < 0.01, η^2^ = 0.54). The test stimulus elicited a significant increase in [HbO] during depth perception compared to the control image (p < 0.001, 99.99 % CI [0.008–0.294]). The similarity between the two stimuli may have resulted in the HDR of the occipital cortex showing no significant increase or decrease of cerebral oxygenation levels during depth stimulation. Cerebral oxygenation measures of [HbO] confirmed the strong association of the right parieto-occipital cortex with processing depth perception. Our study demonstrates the validity of fNIRS to investigate [HbO] and [HbR] during high-level visual processing of complex stimuli.

**Electronic supplementary material:**

The online version of this article (doi:10.1007/s10548-016-0476-4) contains supplementary material, which is available to authorized users.

## Introduction

Binocular vision allows the visual system to fuse each of the 2D images from our retinas by using the difference between them to estimate relative depths. This is termed binocular disparity and is a powerful cue that enables us to achieve depth perception. Research has utilised random dot stereo-pairs or stereograms (RDS) to isolate the effect of binocular disparity, which triggers the perception of depth. RDS stimuli present a pair of images, one to each eye, which when viewed binocularly produce a fused strong percept of depth (Julesz 1971). This percept can be dramatically enhanced by the use of a dynamic stimulus. Consequently, there are individuals who are unable to resolve static RDS to perceive depth, but successfully perceive dynamic RDS (Fujikado et al. 1998; Watanabe et al. 2008). Similarly, behavioural research has shown better psychometric performance when using dynamic RDS compared to static RDS (Allison and Howard 2000; Norman et al. 2006). Yet, static, dynamic RDS and numerous other types of stereoscopic stimuli have been shown to similarly activate large areas of the visual cortex (Parker 2007).

Previous physiological and neuroimaging literature with macaques and humans has identified depth perception to be associated with increased activity in multiple parieto-occipital regions (Herpers et al. 1981; Likova and Tyler 2007; Livingstone and Hubel 1988; Norcia and Tyler 1984; Preston et al. 2008; Shikata et al. 1996; Tanabe et al. 2005; Uka and DeAngelis 2004) compared to the primary visual cortex (Cumming and Parker 1997; Neri et al. 2004; Prince et al. 2002). This high-level visual processing undoubtedly involves numerous cortical areas. These include Brodmann area 19 (Baecke et al. 2009; Fortin et al. 2002; Iwami et al. 2002; Nishida et al. 2001; Thiyagesh et al. 2009), V3a (Backus et al. 2001; Bridge and Parker 2007; Chandrasekaran et al. 2007; Cottereau et al. 2014; Fang and He 2005; Georgieva et al. 2009; Gilaie-Dotan et al. 2002; Goncalves et al. 2015a; Tsao et al. 2003), intraparietal sulcus (IPS) (Baecke et al. 2009; Buckthought and Mendola 2011; Durand et al. 2009; Fang and He 2005; Negawa et al. 2002; Tsao et al. 2003), dorsal V4 (Brouwer et al. 2005; Iwami et al. 2002; Negawa et al. 2002; Tsao et al. 2003), V5 (Brouwer et al. 2005; Chandrasekaran et al. 2007; Cottereau et al. 2014; Fortin et al. 2002; Freeman et al. 2012; Negawa et al. 2002; Orban et al. 2006; Welchman et al. 2005), V6 (Cardin and Smith 2011), and the lateral occipital complex (LOC) (Brouwer et al. 2005; Cottereau et al. 2014; Freeman et al. 2012; Read et al. 2010; Welchman et al. 2005). Dynamic RDS have been reported to elicit responses in similarly numerous regions of interest (ROI) with pronounced activation to depth in the superior parietal lobe (SPL), inferior parietal lobe (IPL) and intraparietal sulcus (IPS) within Brodmann area 7 (Iwami et al. 2002; Thiyagesh et al. 2009), pericalcarine area (Gonzalez et al. 2005), V5 (Smith and Wall 2008), V6 (Cardin and Smith 2011), and the parietal–occipital junction (Paradis et al. 2000; Tyler et al. 2006). Brodmann area 19 has also been associated with dynamic depth perception, specifically area V3a (Iwami et al. 2002; Paradis et al. 2000) and the fusiform gyrus (Gonzalez et al. 2005; Iwami et al. 2002). Due to the size of Brodmann area 19, and the spatial limitations of our neuroimaging technique, we focussed on this area aiming to capture a neurovascular response to depth perception.

The current study utilises functional near infrared spectroscopy (fNIRS), a non-invasive optical neuroimaging technique that has not been used previously to investigate dynamic depth perception. NIRS measures changes of concentrations of blood oxy- ([HbO]) and deoxy-haemoglobin ([HbR]), monitoring the haemodynamic response (HDR) of neuronal stimulation (Villringer et al. 1993). Our own and previous research has used fNIRS to successfully characterise the cortical HDR to simple visual stimuli proving it to be a reliable neuroimaging technique (McIntosh et al. 2010; Toronov et al. 2007; Ward et al. 2015; Wijeakumar et al. 2012b). To expand this, we employ a complex visual stimulus, which uses binocular disparity to induce depth perception and involves high-level neural processing. By employing fNIRS with healthy young adults we measured absolute changes of [HbO] and [HbR] in response to dynamic depth perception.

## Methodology

### Participants

We recruited 13 healthy young adults (mean age 23 ± 4 years, range 18–30, 11 females). All participants had a visual acuity (VA) of at least 6/6 with optical correction where required, and had no history of neurological or psychological disorders. Participants completed a demographic and health questionnaire, had comparable levels of education and were all right handed. Inclusion was based on having stereoacuity within normal clinical limits (Bohr and Read 2013), assessed using the Frisby Near Stereotest. The Frisby test is a measure of ‘real’ depth perception as differences are due to physical depth and no glasses need to be worn (Leat et al. 2001). Participants have to identify the pattern that looks ‘different’ in the four quadrants of a Perspex plate. All participants had good stereoacuity correctly finding the target-in-depth for all plates, with an average stereoacuity of 40 s of arc. Glasgow Caledonian University’s Ethics Committee approved the research protocol, and informed written consent was obtained from all participants prior to testing in accordance with the Declaration of Helsinki.

### Visual Stimuli

**Fig. 1:**
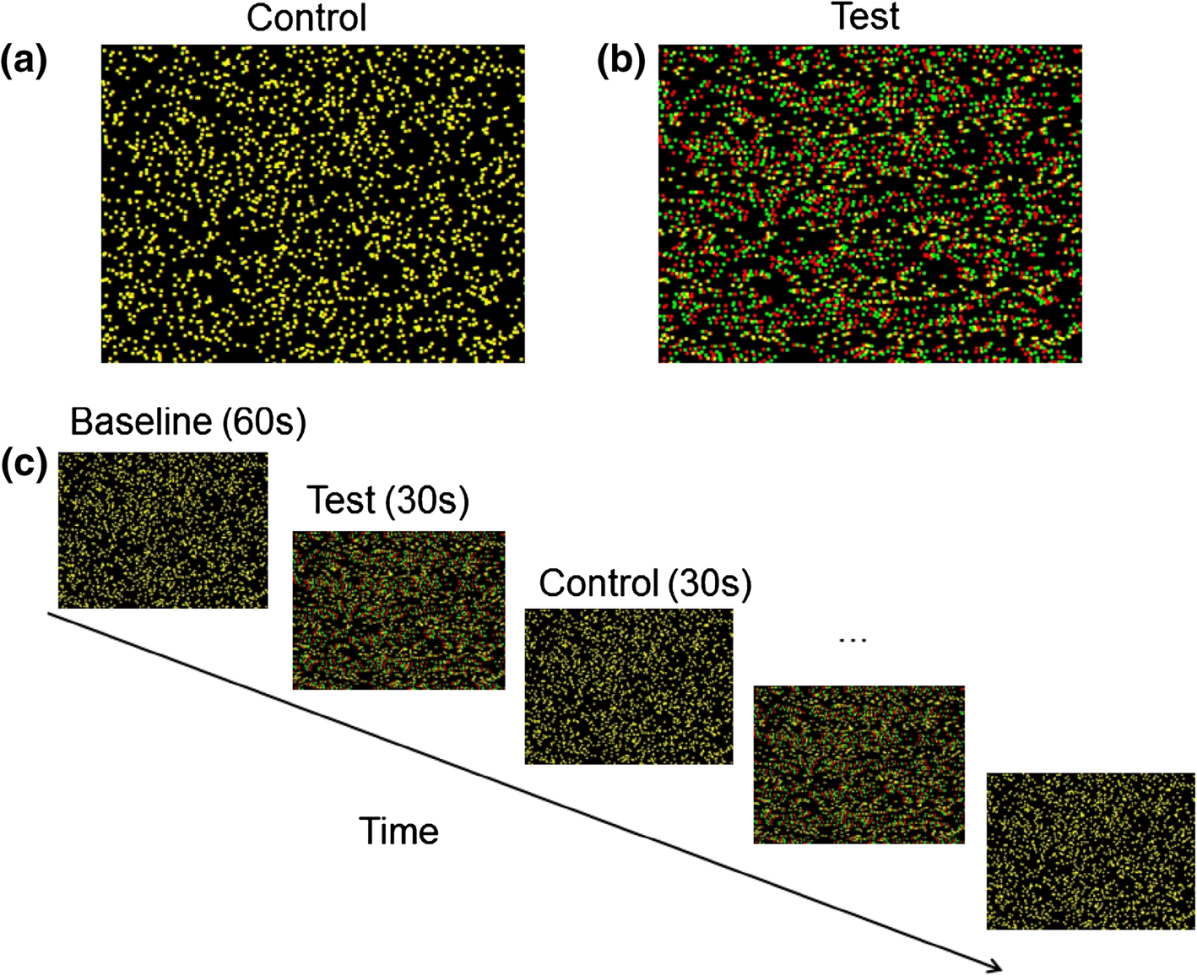
Image of the depth stimuli (dynamic RDS) to be viewed with *red*–*green* anaglyph glasses. **a** Shows the fused ‘flat’ control stimulus (dynamic RDS with zero disparity, perceived as ‘flat’) and **b** is the test stimulus (dynamic RDS with binocular disparity, induced depth percept of 3D vertical sinusoidal waves). **c** Sequence of experimental protocol timings between the conditions (Color figure online)

To investigate depth perception, we manipulated binocular disparity, creating a test image with disparity and a control image without. Consequently, in this study ‘depth’ refers to this manipulation of horizontal binocular disparity within the RDS. To induce depth perception, two variations of dynamic RDS were presented; the test stimuli (Fig. 1b) and control stimuli (Fig. 1a), with, and without depth, respectively. The test stimuli contained horizontal binocular disparity; within the moving dots, a vertical 3D sinusoidal wave would ‘pop out’. From the peak to the trough of this simulated wave pattern, the disparity amplitude was 11 min of arc and 3 sine waves were presented per screen. The control stimulus had zero disparity and the red and green dots appeared superimposed and therefore as a ‘flat’ surface. During testing, participants wore red-green filtered glasses providing each eye a selective view of the colour of the respective dots to the filter. Throughout the experiment, participants fixated on a stationary cross at the centre of the screen. A viewing distance of 1 m was used (visual field size of 20.7° × 15.4°) and the luminance of stimuli was maintained at 0.5 and 16 cd/m^2^.

### Experimental Protocol

We utilised a block design with a single event of 60 s baseline recording in response to the control image (dynamic RDS with zero disparity, perceived as ‘flat’). Following baseline, participants viewed the test stimulus (dynamic RDS with binocular disparity, induced depth percept) followed by the control stimulus (dynamic RDS with zero disparity, perceived as ‘flat’) for 30 s each, 10 times (completing 10 cycles of experimental data). Therefore, the baseline and control stimuli were identical. This paradigm is depicted in Fig. 1d. By employing identical images for the baseline phase and the control cycles of testing we ensured there would not be a generalised visual onset response (Odom et al. 2010).

### HDR Recording

The fNIRS system used was the two-channel frequency-domain multi-distance (FDMD) tissue oximeter (OxiplexTS™). This system is frequency modulated at 110 MHz and data points are sampled at 1 Hz. It uses 2 wavelengths of light (690 and 830 nm) to capture both [HbO] and [HbR]. Along with the photon absorption, scattering and phase information, data are subsequently used to determine accurate and absolute quantification of changes in cerebral oxygenation. FDMD-fNIRS calculates changes in regional haemoglobin concentration in the cortex in absolute concentration (μM/L). This instrumentation has been described in detail elsewhere (Fantini et al. 1995; Gatto et al. 2006; McIntosh et al. 2010). In order to capture the HDR to depth-inducing stimuli, we recorded fNIRS over the parieto-occipital cortex (PO3, PO4) which approximates to Brodmann area 19 (Koessler et al. 2009). Additionally, measurements over the primary visual cortex (V1) were also carried out with recordings over O1 and O2 according to the EEG 10-20 International System of Electrode Placement (Jasper 1958). Participants completed the stimulus block twice with fNIRS measurements recorded in a counterbalanced randomised order.

### Data Analysis

Data pre-processing was completed with a custom-written MATLAB script. All data were normalised with respect to the pre-stimulus baseline using a simple subtraction method. This calculation was carried out according to the most stable response to the control image: an average of the 20 s prior to stimulation. This normalisation procedure addresses individual cerebral oxygenation fluctuations at rest. A moving average low-pass filter was applied with a window size of 8 data points and all data was then detrended. These processes removed potential signal drift and reduced any noise in the data. The HDR to the stimuli was calculated by averaging across all data responses to test and control stimuli cycles. In order to capture *only* depth perception, we subtracted each post-test response from the previous control cycle, thereby removing any effects of stimuli movement. This difference score was used to compare the effect of depth to the baseline measure (control image of dynamic RDS with zero disparity, perceived as ‘flat’). A grand average was taken of the last 15 s of both the difference scores and baseline, representing the greatest stable change of the HDR (McIntosh et al. 2010; Ward et al. 2015; Wijeakumar et al. 2012a, b. This data analysis procedure ensured all parametric assumptions were met and the grand averages were used for inferential statistics, namely repeated measures ANOVA’s and post hoc paired samples t-tests. As there was significant individual variation in the HDR, we additionally Z-transformed participants’ average HDR for the parieto-occipital recordings to determine the importance of this variability. This MATLAB function centres the individual’s responses to have a mean of 0 and a standard deviation of 1, thereby providing a standardised approach for group comparisons across hemispheres.

## Results

### Stimulation Effect

**Fig. 2:**
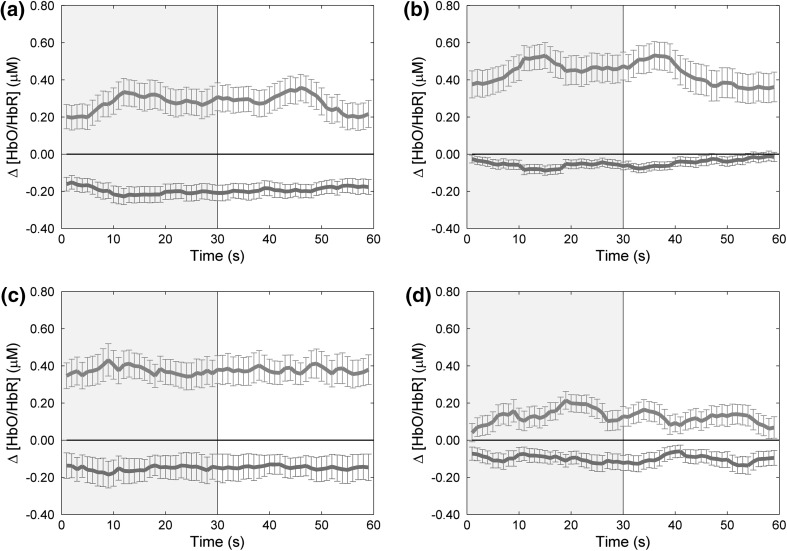
Average HDR to the test stimulus (dynamic RDS with binocular disparity, induced depth percept, *grey area*), and the control ‘flat’ stimulus (dynamic RDS zero disparity, perceived as ‘flat’, *white area*) for the occipital and parietal–occipital cortices (**a** O1, **b** O2, **c** PO3, **d** PO4). [HbO] plotted in *red* and [HbR] in *blue*, mean ± SEM (Color figure online)

There was a clear response to the depth-inducing dynamic RDS in all subjects, although with a great deal of heterogeneity. When examining the group average parieto-occipital (PO3, PO4) HDR to the test stimulus, it can be seen that the right parieto-occipital hemisphere produced a characteristic increase in [HbO] and decrease in [HbR] during depth stimulation compared to the control image (Fig. 2d), whereas the left did not (Fig. 2c). PO3 produced a greater amplitude of response compared to PO4. However, our interest here was not the amplitude of the HDR but the changes between the control and test stimulus conditions. Occipital (O1, O2) HDR produced a fluctuating signal with a bimodal response (Fig. 2a, b); indicating that perhaps each test and control stimuli generated an independent HDR. We attribute this lack of response to binocular disparity specifically, to the comparison between the stimuli, both of which induce a complex visual percept processed in V1. Due to the lack of findings for the occipital data, our results focus on parieto-occipital HDRs only.

**Fig. 3:**
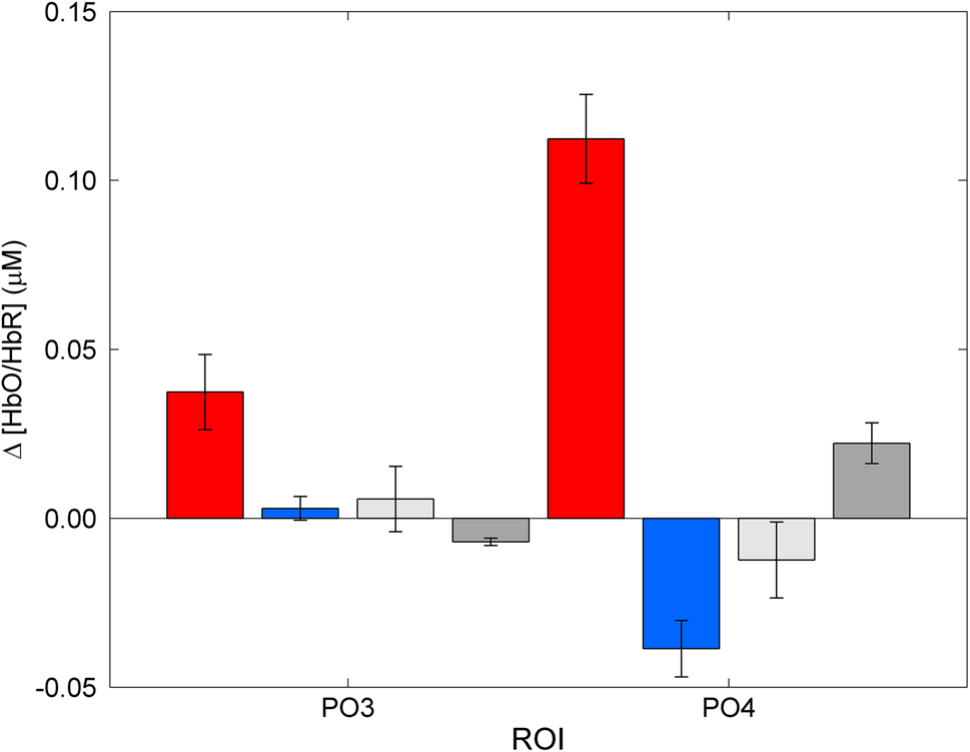
Parieto-occipital grand average cortical responses comparing the test image during depth perception responses ([HbO] plotted in *red*, [HbR] in *blue*) to the control image at baseline ([HbO] plotted in *dark grey*, [HbR] in *light grey*). Means and SEM plotted. Significant differences between depth perception and baseline found for PO4 [HbO] only at p < 0.001 (Color figure online)

Grand average group data were submitted to a median absolute deviation (MAD) outlier analysis and removal was applied. The MAD method provides a robust statistical approach for the removal of outliers relying on median values (Pernet et al. 2013). To examine the effect of stimulation and cerebral oxygenation, separate 2 (induced depth difference score, control stimulus at baseline) × 2 (HbO, HbR) repeated measures ANOVA’s were conducted for each ROI (PO3, PO4, O1, O2). To reduce the likelihood of a Type 1 error (Wilcox 2005), alpha levels were adjusted to provide 98.75 % confidence intervals (CI). Only right hemisphere parieto-occipital recordings produced a significant result, with no stimulation effects in the remaining ROIs. Over PO4, there was a main effect of depth stimulation (F_1,12_ = 13.90, p < 0.01, η^2^ = 0.537), and an interaction between depth stimulation and cerebral oxygenation (F_1,12_ = 22.61, p < 0.001, η^2^ = 0.655). Pairwise comparisons indicated greater [HbO] compared to [HbR], and an increase of cerebral oxygenation during depth stimulation compared to baseline (p < 0.05). To determine where this difference occurred, an adjusted Bonferroni corrected (p < 0.01) post hoc paired samples *t* test was run calculating Cohen’s d_av_ effect sizes for within-subject designs (Cumming 2012; Lakens 2013). Compared to the control image shown at baseline, depth stimulation induced a significant increase in [HbO] (t_12_ = 6.0, p < 0.001, 99.99 % CI [0.008–0.294], Cohen’s d_av_ = 6.85) and a non-significant result for [HbR] (Fig. 3). These results indicate that there is a strong likelihood that PO4 [HbO] increases significantly during binocular disparity compared to fused ‘flat’ stimuli, regardless of inter-subject variability. Although a similar trend can be seen in PO3, the variance in the data perhaps prevented statistical significance.

**Fig. 4:**
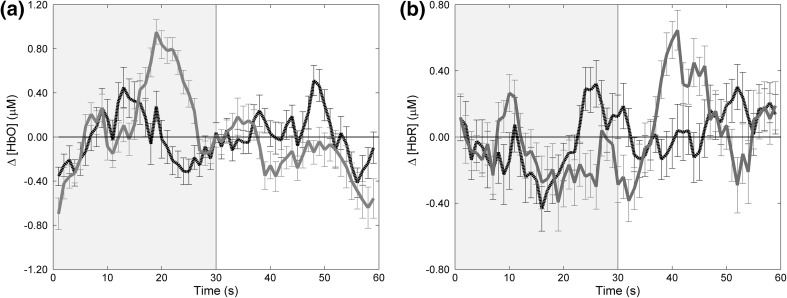
Group averaged Z-transformed HDR of **a** [HbO] and **b** [HbR]. Test stimulus (dynamic RDS with binocular disparity, induced depth percept) in *grey area*, and control ‘flat’ stimulus (dynamic RDS, perceived as ‘flat’) in *white area*, for the parieto-occipital regions (PO3 *black dashed line*, PO4 *red/blue solid line*). Means and SEM plotted (Color figure online)

To examine this variation in the data we Z-transformed participants’ average HDR for the parieto-occipital recordings providing a standardised approach. Based on the above findings, we present only PO3 and PO4 HDR for the Z-transformed group average (Fig. 4). Parietal–occipital cortical responses differ between hemispheres and inter-subject variability within this data is evident with large error bars. Individual Z-transformed average HDR can be found in the supplementary material. There is a great deal of heterogeneity within observers, which may be due to the nature of the binocular disparity within the images used. Yet, Fig. 4 shows that compared to the control ‘flat’ image, PO4 [HbO] increased in response to depth stimulation (test image of depth inducing 3D sinusoidal wave).

## Discussion

The current study used fNIRS to measure the HDR associated with depth perception in response to a dynamic depth stimulus. Reliable visual stimulation effects were seen in the right parieto-occipital hemisphere wherein there was a characteristic increase of [HbO] and decrease of [HbR] during presentation of the test stimulus (horizontal disparity, induced depth percept), compared to the control stimulus (zero disparity, perceived as ‘flat’). Our data concurs with and expands on BOLD evidence that relates predominantly to [HbR] (Fabiani et al. 2014; Mehagnoul-Schipper et al. 2002; Näsi et al. 2010; Rees et al. 1997). In the parieto-occipital cortex, we report coupling in cerebral oxygenation with a mirrored image between [HbO] and [HbR] during depth perception (Fig. 2c). An additional significant finding was that of a hemispheric dominance effect with the right hemisphere producing statistically significant changes in [HbO] compared to the left which produced a bimodal HDR (Fig. 2d). Our results contribute to the literature supporting a right hemisphere bias in depth perception (Baecke et al. 2009; Durnford and Kimura 1971; Hirsch et al. 1995; Nishida et al. 2001; Taira et al. 2001) directly contradicting evidence relating to no depth perception lateralisation (Backus et al. 2001; Buckthought and Mendola 2011; Fang and He 2005; Lehmann and Julesz 1978; Mendola et al. 1999; Merboldt et al. 2002; Tsao et al. 2003). This hemispheric dominance controversy is no doubt fuelled by the extent of heterogeneity both in previous research and the current dataset. In Fig. 4 this variability is highlighted and can be seen as Z-transformed group averaged HDRs plotted from both parieto-occipital cortices. Previous research has similarly shown such variation, for example, Huppert et al. (2006) report fNIRS findings with considerable inter-subject variability in the shape and timing of the HDR relating to motor activity. With respect to complex visual stimulation and fMRI, Baecke et al. (2009) describe varied data with less than half of their participants demonstrating a right hemisphere bias in depth perception. The authors stress that the inconsistency of results relating to hemispheric dominance may be masked in smaller sample sizes. Therefore, the current results presenting absolute values of [HbO] in the right but not left parieto-occipital cortex in response to induced depth are compelling.

Although the left parieto-occipital cortex appeared to have a trend in the dataset, the lack of statistical response need not be surprising. A previous unpublished fNIRS study in a thesis by Wijeakumar (2011) reports a similar bimodal HDR for PO3, as well as the primary visual cortex (V1), in response to dynamic RDS. We propose the variance and small sample size to potentially occlude results of depth processing in PO3. Our V1 recordings showed responses to both stimulus images, with no significant HDR to depth perception. It can be argued that V1 HDR present as bimodal signals with V1 activity relating to both of the complex stimuli, regardless of the difference between the images in binocular disparity. Although V1 contains both binocularly and monocularly activated cells and is involved in depth processing, evidence indicates this specialisation occurs higher in the visual pathway. Macaque studies have proposed individual V1 neurons are not selective for conscious processing of stereoscopic depth (Cumming and Parker 1997). It is now widely accepted that single V1 cells generate the cascade of higher-level processing where depth is fully perceived (Herpers et al. 1981; Hirsch et al. 1995; Iwami et al. 2002; Merboldt et al. 2002; Rees et al. 2002). Indeed, high resolution fMRI imaging (7T) has shown that although V1 does show cortical activation in response to binocular disparity, it is not to the same extent as V3A. This is shown to be consistent regardless of the width of disparity used, and Goncalves et al. (2015a) conclude that activity in area V3A relates directly to the reported perceptual discrimination thresholds of binocular disparity images. High resolution imaging provides promising insights into V1 processing of stereopsis as recent work has suggested it is the deep layers of V1 that show a preference for binocular disparity (Goncalves et al. 2015b). These findings are in accordance with the current proposed feedback mechanisms between cortical areas within the visual system.

A similar study with fNIRs and static RDS found responses to both occipital and parietal–occipital cortices to depth perception (Wijeakumar et al. 2012a). However, Wijeakumar et al.’s control stimulus image was a black screen, therefore the test stimuli was a ‘novel’ response with the consequences of the first presentation always eliciting an onset response. In the current study, both control and test stimuli were identical with the exception that the test RDS had a shift in the dots that induced binocular disparity, therefore capturing the response made specifically to depth. However, both studies (Wijeakumar et al., and the current one) found a parieto-occipital HDR to depth perception. This supports the notion of shared neural correlates of depth perception between types of stimuli, as previously discussed. Additional support for this comes from a fMRI study on humans and macaques in which both static and dynamic RDS activated the same ROI (Tsao et al. 2003). Furthermore (Gonzalez et al. 2005) report similar ROI for both types of depth stimuli in recordings of subdural electrode VEPs in a 47-year old woman. Multimodal imaging has provided us with valuable insights regarding this integration between the vascular HDR and the neural electrical counterpart, i.e. neurovascular coupling (Fabiani et al. 2014; Iwaki et al. 2013). Iwaki and colleagues combined data from both fMRI and MEG and describe the parieto-occipital, intraparietal and posterior infero-temporal regions to be active during perception of 3D objects from moving dots. Further multimodal or combination neuroimaging studies will enlighten this seemingly complex relationship between the neural and vascular responses of the brain regarding depth perception.

Although this study has inherent limitations of one NIRS channel per hemisphere of recording, and a small sample, we report absolute values of [HbO] and [HbR] and therefore oxygenation, in response to depth perception in healthy young adults. An avenue of future work would be to use the same depth stimuli with EEG in order to couple the data with fNIRS, combining two approaches. We also intend to examine the HDR in participants with limited stereoacuity, e.g. amblyopia. Individuals with amblyopia may have reduced or absent stereoacuity, therefore we hypothesise that they would present with an attenuated HDR in the parieto-occipital cortex. Similar results have been presented using EEG where patients with reduced stereoacuity (mostly microstrabismus) have higher VEP thresholds to RDS, particularly in the right visual field (Skrandies 2009).

In conclusion, we have successfully recorded the HDR associated with dynamic depth perception using fNIRS. Healthy young adults showed a characteristic increase of [HbO] and decrease of [HbR] during complex visual stimulation in the parieto-occipital cortex. In line with previous neuroimaging work, we report a HDR over right hemisphere Brodmann area 19 for processing depth perception, with a large effect size. Occipital recordings fluctuated due to the complexity of both the test and control images. Within our young adult sample there was a strong coupling between [HbO] and [HbR]. Our study demonstrates that fNIRS is a suitable technique to investigate the HDR during high-level visual processing of complex stimuli.

## Electronic supplementary material

Below is the link to the electronic supplementary material.

Supplementary material 1 (DOCX 664 kb)
