# Supplementary material for: Using Functional Near Infrared Spectroscopy (fNIRS) to Study Dynamic Stereoscopic Depth Perception
Source: Brain Topogr. 2016 Feb 22;29:515–23. doi: 10.1007/s10548-016-0476-4 (PMC4899499; doi:10.1007/s10548-016-0476-4)
Supplement: Supplementary file 1 — Supplementary material 1 (DOCX 664 kb) [file 10548_2016_476_MOESM1_ESM.docx]

**
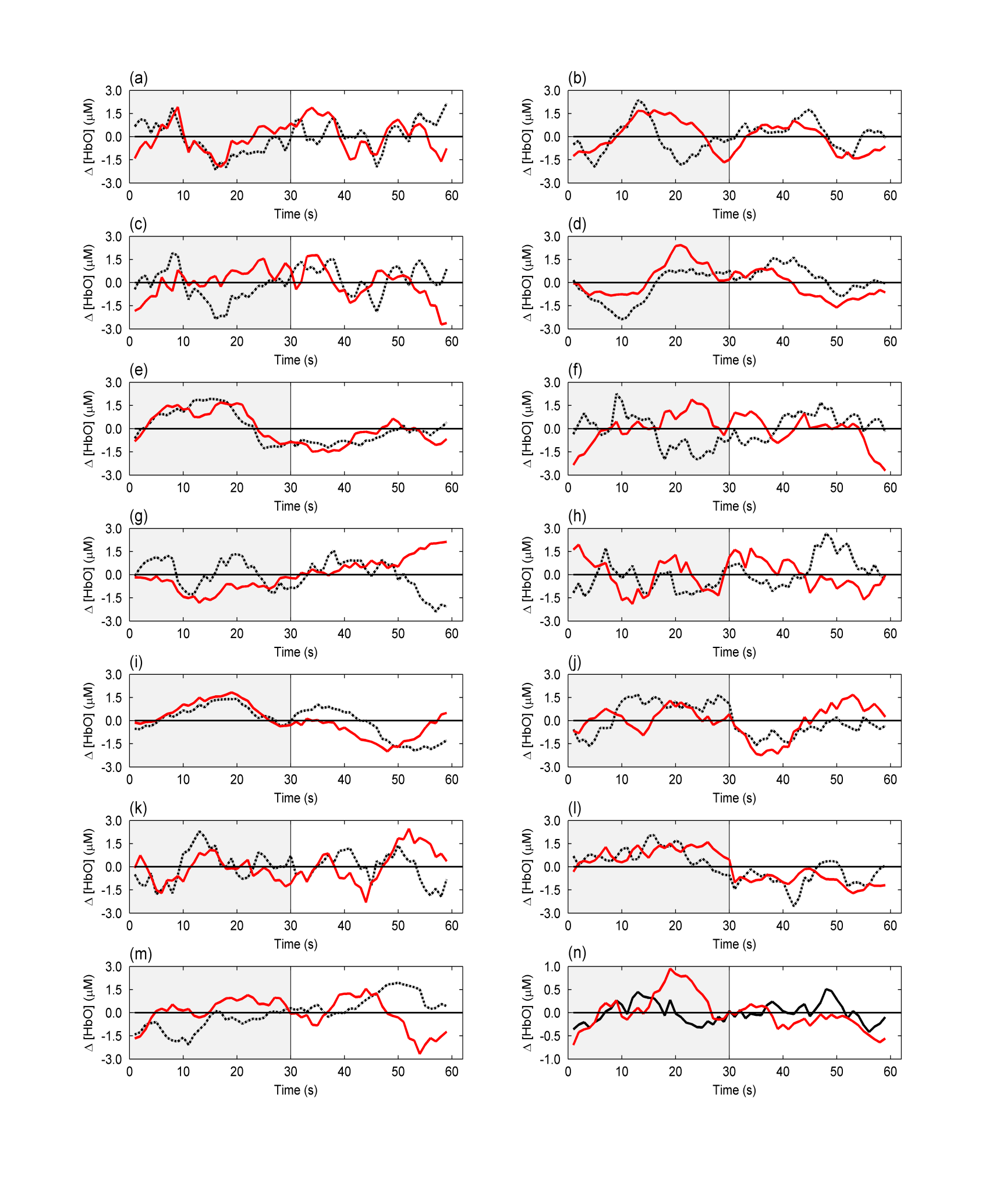
Supplementary Material**

**Fig. 1** (a – m) Individual Z-transformed average HDR of participant’s [HbO], means and s.e.m plotted. Test stimulus of dynamic RDS with binocular disparity, induced depth percept (grey area) and control ‘flat’ stimulus of dynamic RDS with zero disparity (white area) are depicted for the parieto-occipital regions (PO3 black dashed line, PO4 red solid line). (n) Group average Z-transformed HDR.
